# Immunoregulatory protein B7-H3 upregulated in bacterial and viral infection and its diagnostic potential in clinical settings

**DOI:** 10.3389/fimmu.2024.1472626

**Published:** 2024-10-21

**Authors:** Abiye Tigabu

**Affiliations:** Department of Medical Microbiology, University of Gondar, Gondar, Ethiopia

**Keywords:** immunoregulatory protein, B7-H3, diagnosis, bacteria, virus, infection

## Abstract

Bacterial and viral infections cause a huge burden to healthcare settings worldwide, and mortality rates associated with infectious microorganisms have remained high in recent decades. Despite tremendous efforts and resources worldwide to explore diagnostic biomarkers, rapid and easily assayed indicators for the diagnosis of bacterial and viral infections remain a challenge. B7 homolog 3 (B7-H3), a member of the B7 family of immunoregulatory proteins, is overexpressed in patients with septicemia, meningitis, pneumonia, and hepatitis. Therefore, B7-H3 could be used as a potential clinical indicator and therapeutic target for bacterial and viral infections caused by *H. pylori*, *S. pneumoniae*, *M. pneumoniae*, hepatitis B virus (HBV), viral hemorrhagic septicemia virus (VHSV), respiratory syncytial virus (RSV), and human immunodeficiency virus (HIV). Moreover, the interplay between infectious microorganisms and B7-H3 and exploration of the functional roles of the B7-H3 molecule could aid in the development of novel strategies for disease diagnosis and immunotherapy.

## Introduction

Bacterial and viral infections are common in children and adults. Fever is a common symptom of infectious diseases, suggesting systemic inflammation in response to bacterial or viral infections. The non-specific nature of signs and symptoms in febrile patients makes clinical differentiation of infections challenging, particularly in identifying severe diseases such as septicemia, meningitis, and pneumonia. For optimal treatment, early diagnostic biomarkers indicating bacterial or viral infections are required to reduce mortality from serious infections. Despite the difficulty in distinguishing between infection, inflammation, and autoimmunity, biomarkers in combination with the symptoms of the patient support physicians in considering proper diagnosis and treatment (1, 2). Although white blood cell counts (WBCs), C-reactive protein, and inflammatory cytokines are useful diagnostic indicators of infections, more rapid and easily assayed indicators can advance diagnosis (3).

B7 homolog 3 (B7-H3, CD276) is a member of the B7 family of immunoregulatory proteins, sharing 20–27% amino acid identity with other B7 family members (4, 5). It is a type I transmembrane protein that primarily functions as a negative immunoregulatory protein (6). B7-H3 is a novel protein structurally related to the B7 family of ligands, characterized by a single extracellular IgV- and IgC-like domain in the transmembrane region and a highly diverse cytoplasmic tail. The predominant form of human B7-H3, 4IgB7-H3, contains tandemly duplicated IgV-IgC domains due to exon duplication, which generates two isoforms: 2IgB7-H3 and 4IgB7-H3. Additionally, serine- and arginine-rich splicing factor 3 (SRSF3) plays a role in the splicing of B7-H3 by binding directly to exons 4 and/or 6 (7–10).

## B7-H3 expression and its impact on tumor immune microenvironment

Several studies have shown that B7-H3 can be detected in immune cells, such as activated macrophages, dendritic cells, monocytes, myeloid-derived suppressor cells, activated T cells, and various types of cells in non-lymphoid tissues, including tumor cells, epithelial cells, fibroblast-like synoviocytes, osteoblasts, and human serum. B7-H3 is overexpressed in tumor tissues and shows limited expression in normal tissues (6, 11, 12). Confocal microscopy of fibroblast-like synoviocytes and T-cell co-cultures showed B7-H3 localization at the T-cell–fibroblast-like synoviocyte contact point (13). Most human intratumoral neutrophils express high levels of B7-H3, and locally enriched B7-H3^+^ neutrophils are positively correlated with increased granulocyte-macrophage colony-stimulating factor levels (14).

B7-H3 is an immunoregulatory ligand that affects immune responses through both immunological and non-immunological pathways (15) and exerts either inhibitory or stimulatory effects on immune cell activation (16). Activated CD4^+^ and CD8^+^ T cells express a putative receptor that recognizes B7-H3 molecules (17). The expression of B7-H3 favors an immunosuppressive microenvironment by promoting IL-10 and TGF-β1 (18). The VC and VCVC forms of human B7-H3 inhibit CD4^+^ T cell activation, proliferation, and cytokine production (19), as well as attenuate NK cell-mediated killing (20). B7-H3 expression inhibits the activation of CD4^+^ T cells, CD8^+^ T cells, γδT cells, CAR-T cells, Vδ2 T cells, Th17 cells, CD3^+^ T cells, macrophages, neutrophils, and dendritic cells, as well as the secretion of IFN-γ, IL-2, and perforin/granzyme B (21–24).

B7-H3 regulates the differentiation of tumor-associated macrophages, promotes the polarization of type 2 macrophages, and switches the M1 phenotype to the M2 phenotype (25). B7-H3 recruits macrophages into the tumor microenvironment (26) and contributes to CCL2–CCR2–M2 macrophage axis-mediated immunosuppression (27). Zhou et al. reported that the high expression of B7-H3 in human prostate cancer tissues is negatively correlated with CD8^+^ tumor-infiltrating lymphocytes (28). However, some studies have reported that human patients with high B7-H3 expression show increased numbers of immune cells, including CD8^+^ T cells, CD4^+^ T cells, natural killer cells, plasmacytoid dendritic cells, and increased interferon-γ production (4, 29, 30).

## The role of B7-H3 in modulating immune responses during bacterial infections

Several studies have shown that microorganisms have a profound impact on many aspects of cell function and are involved in many diseases (31–33). An infection happens when bacteria, viruses, or fungi invade the body, and damage the host. While the immune system works to eliminate these invaders, sometimes the pathogens overpower the body’s defenses, resulting in illness (34). The gut microbiota plays a crucial role in modulating the immune system. However, its dysbiosis induces chronic inflammation. For example, *E. coli* and *B. fragilis* are known to promote inflammation in the gut that leads to DNA damage and tumor formation (35). In mouse model, a microbiota-dependent pathway crosstalk between myeloid cells, T cells, and tumor cells that inhibits CD8^+^ T cell-dependent anti-tumor immunity through the co-inhibitory protein B7-H3 (34).

Bacterial sensing by myeloid cells promotes calcineurin- and NFAT-dependent IL-6 release. This IL-6, in turn, promotes the expression of co-inhibitory B7-H3 by tumors, which inhibits CD8^+^ T cell-dependent antitumor immunity, whereas B7-H3 blockade elicits protective T cell responses (36). *Helicobacter pylori* infection induces B7-H3 expression in human gastric epithelial cells through the type 4 secretion system components, CagA, and cell wall peptidoglycan fragments. These are recognized by the intracellular pattern recognition receptor NOD1, which activates the MAPKs and NF-κB pathways. During *H. pylori* infection, patients exhibit a mixed Th1/Th2 response, with increased circulating Treg and Th17 cells. Human biopsy samples from patients with gastritis and gastric tumors show increased B7-H3 expression and Th2 responses in *H. pylori* strains associated with gastritis (37).

B7-H3 functions as a costimulatory molecule in innate immunity by augmenting the release of proinflammatory cytokines from monocytes and macrophages stimulated by bacterial cell wall products, contributing to the development of sepsis. B7-H3 enhances human sepsis through bacterial lipopolysaccharide (LPS)- and lipoprotein-induced NF-κB activation and inflammatory responses. However, blocking B7-H3 *in vivo* attenuated LPS-induced proinflammatory cytokine release and reduced endotoxin shock-related lethality. Furthermore, human patients diagnosed with sepsis exhibit significantly higher levels of plasma soluble B7-H3 than healthy individuals. Stimulation of human monocytes with LPS and inflammatory cytokines leads to substantial release of soluble B7-H3 (38).

Circulating B7-H3 levels in cerebrospinal fluid (CSF) and plasma were higher in children with bacterial meningitis than in the control group. Additionally, circulating TNF-α levels in CSF and plasma were higher in the bacterial meningitis group than in the control group. On admission, circulating B7-H3 levels in the plasma and CSF of patients with bacterial meningitis were positively correlated with TNF-α, IFN-γ, and white blood cell counts, making them useful markers for distinguishing bacterial from aseptic meningitis and for evaluating the intensity of the infectious inflammatory process in the central nervous system (39).

The costimulatory protein B7-H3 contributes to the development and progression of pneumococcal meningitis by augmenting the innate immunity-associated inflammatory response in a TLR2-dependent manner. B7-H3 enhances the formation of the MyD88-IRAK immunocomplex in the brains of *S. pneumoniae*-infected mice and significantly augments *S. pneumoniae*-induced activation of TLR2 downstream of the NF-κB p65 and MAPK p38 pathways. This exacerbates mouse brain damage by intensifying inflammatory responses (40, 41).

B7-H3 plays a role in *S. pneumoniae* infection-induced pneumococcal meningitis by amplifying the inflammatory response, worsening blood-brain barrier disruption, and aggravating the clinical disease status via a TLR2-dependent mechanism. B7-H3 augments proinflammatory cytokine and chemokine production, upregulates NF-κB, TLR2, p65, and MAPK p38 phosphorylation, and enhances the nuclear transactivation of NF-κB p65 at the TNF-α and IL-6 promoters in *S. pneumoniae*-stimulated microglial cells of the mice (42).

Soluble B7-H3 levels were significantly higher in human patients with *M. pneumoniae* pneumonia (MPP) compared to control subjects. Furthermore, soluble B7-H3 plays an important role in MPP by increasing TNF-α concentrations and neutrophil activation (43). Elevated levels of soluble B7-H3 were found in both mild and severe MPP in pediatric patients compared with control patients. Moreover, significantly higher levels of soluble B7-H3 were detected in patients with severe MPP compared with those with mild MPP. The receiver operating characteristic (ROC) curve showed that soluble B7-H3 had a severity prediction capacity for mild and severe MPP and was positively associated with IFN-γ and GM-CSF in patients with severe MPP. Additionally, elevated levels of soluble B7-H3 were found in acute-phase MPP patients compared with control subjects, while significantly lower levels of plasma soluble B7-H3 were observed in recovery-phase MPP patients compared with acute-phase patients (44).

Children with MPP and pleural effusion had higher levels of soluble B7-H3 and IL-36 than control subjects. The concentration of soluble B7-H3 in bronchoalveolar lavage fluid was strongly associated with IL-36 levels, the duration of fever, and length of hospital stay (45). Additionally, children with MPP had higher levels of soluble B7-H3 and IL-17 than controls, especially during the acute stage of MPP. Children with MPP and pleural effusion had higher levels of soluble B7-H3 than those without pleural effusion, and these levels were positively correlated with the number of fever days (46). During *H. pylori*, *S. pneumoniae*, and *M. pneumoniae* infections, elevated B7-H3 levels were detected in human biopsy, CSF, and serum samples. The interplay between bacteria and B7-H3 expression in different disorders has been documented in several studies (36–41, 43, 44) and the results are presented in **Table 1**.

**Table 1:** The interplay between bacteria and B7-H3 expression in different disorders, 2024.

Disorders	Specimens	Methods	Causative agent	Interaction of bacteria and B7-H3 expression
**Gastritis**	Gastric biopsies	Culture, flow cytometry, PCR	*H. pylori*	*H. pylori* uses a type 4 secretion system and cytokines produced by Th17 and Treg cells to upregulate B7-H3 expression via the p38 MAPK pathway and then inhibit T cell response (37).
**Colorectal cancer**	Colon tissue	Western blot, 16S rRNA sequencing	Gut microbiota	Gut microbiota promotes calcineurin and NFAT-dependent IL-6 release, and NFAT-dependent IL-6 promotes expression of B7-H3 by tumors and it inhibits CD8^+^ T cell-dependent anti-tumor immunity (36).
**Sepsis**	Blood/plasma	Culture, ELISA	Bacterial LPS and lipoprotein	B7-H3 functions as a costimulatory of innate immunity by augmenting proinflammatory cytokine release from bacterial product stimulated monocytes/macrophages (38).
**Meningitis**	Brain tissue, plasma, CSF	Culture, PCR, ELISA	*S. pneumoniae*	*S. pneumoniae* induces phosphorylation of NF-κB p65, MAPK p38, and ERK1/2 pathways, TNF-α, and substantially augments B7-H3 expression (39–41).
**Pneumonia**	NPA, Plasma	Culture, PCR, ELISA	*M. pneumoniae*	B7-H3 increases immunopathogenesis of *M. pneumoniae* pneumonia by increasing TNF-α, IFN-γ, GM-CSF concentration, and activation of neutrophils (43, 44).

PCR, Polymerase chain reaction; ELISA, Enzyme-linked immunosorbent assay; LPS, Lipopolysaccharides; CSF, Cerebrospinal fluid; NPA, Nasopharyngeal aspirate.

## B7-H3 as a modulator of immune responses in viral infections

Costimulatory molecules are important regulators of the immune response and participate in the regulation of liver pathology during hepatitis B virus (HBV) infection. The costimulatory protein B7-H3 is upregulated after HBV infection and contributes to the progression and poor prognosis of HBV infection by triggering inhibitory signals in effector T cells. The membrane and soluble forms of B7-H3 are expressed on Treg cells and monocytes and are positively correlated with the frequency of Treg cells in patients with acute hepatitis B (AHB), chronic hepatitis B (CHB), and hepatocellular carcinoma (HCC) associated with HBV infection. Soluble B7-H3 levels are higher in the late tumor-node-metastasis (TNM) stages of HCC. Moreover, B7-H3 expression positively correlates with aspartate aminotransferase and alanine aminotransferase levels in chronic HBV infection. Immunohistochemistry tests show that higher membrane B7-H3 expression is associated with larger tumor size, later TNM stages, and worse prognosis in HBV-HCC (47).

Luan et al. found that abundant plasma-soluble B7-H3 positively correlated with liver fibrosis in children with chronic HBV infection (46). Soluble B7-H3 originates from the hepatocyte membrane and promotes hepatic inflammation and hepatitis progression. A functional study showed that immobilized B7-H3 fusion protein inhibits TCR-induced proliferation and IFN-γ secretion by T cells (48). Immunohistochemical analysis detected B7-H3 in all HBV-related acute and chronic liver failure (HBV-ACLF) human biopsy samples. B7-H3 is found on cell membranes and in the cytoplasm of HBV-ACLF samples, and its expression is predominantly observed in infiltrating inflammatory cells and damaged bile ducts (49). B7-H3 was co-expressed with the herpes virus entry mediator in human liver tissues, and with B and T lymphocyte attenuators and the herpes virus entry mediator (50).

Human respiratory tract epithelial cells express a wide range of B7 molecules, and B7-H3 is strongly expressed in unstimulated tracheal, bronchial, and alveolar epithelial cells. Respiratory syncytial virus (RSV) infection of tracheal, bronchial, and alveolar epithelial cells upregulates B7-H3 expression. The high expression of B7-H3 following RSV infection is regulated by IFN-γ and IL-4, which may be involved in decreasing T cell antiviral immune responses to RSV and RSV-associated wheezing. On RSV-infected alveolar epithelial cells, IFN-γ treatment decreases B7-H3, while IL-4 treatment increases B7-H3 expression (51).

Evidence shows that B7-H3 mRNA is broadly expressed in both lymphoid and non-lymphoid organs. Viral hemorrhagic septicemia virus (VHSV) induces the transcription of B7-H3 in the fish liver during the early hours, and it is expressed later in the fish head, kidney, spleen, intestine, and gill tissues. Flow cytometric analysis of leukocytes revealed that 85.1% of granulocytes and 3.1% of lymphocytes expressed B7-H3 molecules on their cell surfaces. The co-inhibitory molecule B7-H3 participates in regulating cell-mediated immune responses during VHSV infection (7).

Individuals with human immunodeficiency virus (HIV) infection have a higher incidence of various malignancies, and HIV-mediated immune dysfunction may lead to chronic immune activation, decreased tumor surveillance, and subsequently increased cancer risk. HIV-infected patients with lung cancer had significantly higher B7-H3 tumor expression levels than HIV-uninfected controls. B7-H3 expression was 92% in lung cancer samples from HIV-infected cases, compared to 69% in samples from HIV-uninfected cases (52). These studies suggest that B7-H3 may play a role in viral pathogenesis and could offer a promising approach for the diagnosis and treatment of viral infections. Previously, the interaction between different virus and B7-H3 protein has been reported in different diseases (7, 47–49, 51, 52) (**Table 2**).

**Table 2:** The interaction between virus and B7-H3 protein in different diseases.

Disorders	Specimens	Methods	Etiology	Interaction b/n virus and B7-H3 expression
**Hemorrhagic septicemia**	Different tissues	Flowcytometry, PCR	VHSV	VHSV stimulates the transcription of B7-H3 (7).
**Hepatitis**	Biopsies, Plasma	IF, ELISA, IHC, Western blot	HBV	Co-expressed with HVEM and promote liver fibrosis, and pathogenesis by inhibiting T-cell responses (47–49).
**Respiratory infections**	Epithelial cells	Flow cytometry	RSV	RSV infection increases B7-H3 expression and decreases T immune responses to RSV via IFN-γ and IL-4 (51).
**Lung cancer**	Lung tissue	IHC	HIV	HIV-mediated immune dysfunction increases B7-H3 expression and cancer risk (52).

HBV, Hepatitis B virus; HBV-ACLF-HBV, related acute-on-chronic liver failure; VHSV, Viral hemorrhagic septicemia virus; IF, Immunofluorescence; HIV, Human immunodeficiency virus; IHC, Immunohistochemistry; RSV, Respiratory syncytial virus; HVEM, Herpes virus entry mediator.

## Emerging receptors for B7-H3: current candidates and future research directions

The putative receptors for B7-H3 remain under investigation, but several candidates have emerged. The T-cell immunoreceptor with Ig and ITIM domains (TIGIT) has been suggested as an inhibitory receptor that could interact with B7-H3, contributing to immune evasion in tumors (53). The herpesvirus entry mediator (HVEM), another B7 family member, has also been proposed as a potential receptor, potentially influencing T-cell activation (54). Ongoing research is exploring whether B7-H3 might interact with other immune checkpoint receptors, such as PD-1 or CTLA-4 (55). Overall, while several receptor candidates exist, further research is needed to fully elucidate B7-H3’s potential receptors and their broader implications in immune modulation.

## B7-H3 versus traditional markers: a new frontier in early disease diagnosis and treatment

B7-H3 has shown greater specificity to some bacterial and viral infections, particularly in patients with septicemia, meningitis, pneumonia, and hepatitis, thereby reducing the risk of false positives and negatives. Studies indicate that B7-H3 is significantly overexpressed in *H. pylori*, *S. pneumoniae*, *M. pneumoniae*, HBV, VHSV, RSV, and HIV compared to traditional markers, making it more reliable for early detection and disease progression monitoring (56). Unlike some traditional markers, B7-H3 also holds promise as a therapeutic target, opening possibilities for combined diagnostic and therapeutic strategies (57). However, many traditional markers, such as WBCs, C-reactive protein, and cytokines, have lower sensitivity in the early stages of disease, which may delay diagnosis and intervention. Moreover, traditional markers may not provide the same level of specificity in distinguishing between closely related conditions, leading to potential diagnostic ambiguities. While the implementation of B7-H3 in clinical settings is still being refined, early studies suggest that its integration into diagnostic workflows would not significantly increase complexity or cost, particularly with the development of standardized assays. B7-H3’s dual potential as both a diagnostic marker and a therapeutic target offers a long-term advantage (55), potentially streamlining patient care through personalized medicine approaches.

## B7-H3’s role in differentiating diseases with overlapping symptoms

Febrile diseases often share common symptoms such as fever, fatigue, and malaise, which complicates early differentiation between infectious, autoimmune, or malignant conditions. B7-H3’s expression pattern offers a significant advantage in distinguishing between these conditions in the early stages, where other traditional markers may lack specificity. For example, B7-H3’s elevated expression in bacteria, and virus-infected patients allows for earlier detection when other markers remain inconclusive due to overlapping clinical presentations. We also highlight that B7-H3 can improve diagnostic accuracy during the initial symptomatic phase, facilitating prompt and targeted treatment (58, 59). This ability to provide early and reliable differentiation underlines B7-H3’s strength as a promising superior diagnostic tool.

## Challenges and limitations of B7-H3 as a clinical diagnostic biomarker

B7-H3 has several limitations as a clinical diagnostic assay. Its broad expression across various diseases, including cancer, autoimmune disorders, and infections, reduces its specificity, making it difficult to determine if upregulation is caused by a particular pathology or a general immune response (60). Additionally, B7-H3 expression can vary significantly across different cancers and even within cancer subtypes, complicating its use as a reliable marker (61). Its overlap with other immune checkpoint molecules, such as PD-L1 and B7-H4 (62), further challenges the ability to distinguish its specific role. Moreover, the lack of standardized clinical assays and established cut-off values for B7-H3 detection limits its widespread clinical use. B7-H3 is also dynamically regulated by immune signals, which can lead to fluctuating expression levels during disease progression, increasing the risk of inconsistent diagnostic results. Furthermore, its dual role in both immune activation and suppression complicates interpretation, as it may signal different biological processes depending on the context. Finally, while B7-H3 shows promise as a therapeutic target, its predictive value for treatment response remains unclear, limiting its use in guiding therapy decisions. Therefore, B7-H3 would likely need to be combined with other biomarkers to improve diagnostic accuracy.

## Current ongoing clinical trials targeting B7-H3 and their mechanisms

Although B7-H3 has not been extensively studied in bacterial and viral infections, its role in immune suppression may hold potential relevance for future infection-related research. By inhibiting immune checkpoints like B7-H3, it might be possible to enhance the body’s ability to fight not only tumors but also infections. However, at present, there are no ongoing trials directly targeting B7-H3 for bacterial or viral infections. Currently, clinical trials targeting B7-H3 primarily focus on cancer rather than on bacterial or viral infections (63). Several antibody-drug conjugates (ADCs) and chimeric antigen receptor (CAR)-T cell trials exploring therapies targeting B7-H3 for different cancer types are listed in **Table 3**.

**Table 3:** Ongoing clinical trials targeting B7-H3 in cancer therapy.

Trial Name/ID	Therapy type	Cancer types	Phase	Mechanism	Company	Status
**Vobramitamab Duocarmazine (MGC018)**	ADC	Prostate cancer, lung cancer, breast cancer	II	Targets B7-H3-expressing tumors to deliver cytotoxic agents directly to cancer cells	MacroGenics	Ongoing
**Enoblituzumab (MGA271)**	Monoclonal antibody	Head and neck cancer, melanoma	II	Enhances immune response by blocking B7-H3, promoting T-cell-mediated tumor destruction	MacroGenics	Ongoing
**HS-20093**	ADC	Various solid tumors	I/II	Targets B7-H3 to deliver a cytotoxic payload directly to tumor cells	HanchorBio, Hillhouse Capital	Ongoing
**B7-H3 CAR T-cell Therapy**	CAR-T cells	Solid tumors, neuroblastoma	I	Genetically engineered T-cells to target B7-H3 on tumors	Various (U.S. & China)	Ongoing
**Ifinatamab Deruxtecan (m276-SL-PBD)**	ADC	Small cell lung cancer (SCLC)	II	Targets B7-H3, inducing cytotoxicity in cancer cells	Daiichi Sankyo	Active
**DS-7300**	ADC	Advanced solid tumors	I	Binds to B7-H3 on cancer cells and delivers cytotoxic agents to kill them	Daiichi Sankyo	Recruiting

## Conclusion and future perspectives

Taken together, published evidence suggests that B7-H3 might contribute to the progression of bacterial and viral infections by triggering inhibitory signals in effector T cells and is associated with poor prognosis during these infections. Many patients with suspected febrile disease are present with similar or overlapping clinical symptoms, which makes early diagnosis difficult. A novel biomarker for infection in febrile patients is needed to help physicians make the correct diagnosis and initiate appropriate treatment to improve patient outcomes. Herein, we review the discovery of novel protein biomarkers to improve current diagnostics and accelerate early and personalized treatment decisions. Therefore, B7-H3 could be utilized as a potential diagnostic marker in addition to white blood cell counts, C-reactive protein, and inflammatory cytokines, and as a potential therapeutic target against bacterial and viral infections. In-depth studies should be conducted to explore the role of B7-H3 in these infections.
